# The association between different sleep health dimensions and sex, age, education, circadian preference, and chronic insomnia: a representative population-based study

**DOI:** 10.1093/sleepadvances/zpad041

**Published:** 2023-10-27

**Authors:** Bjørn Bjorvatn, Siri Waage, Ståle Pallesen, Daniel J Buysse, Ingvild W Saxvig

**Affiliations:** Department of Global Public Health and Primary Care, University of Bergen, Bergen, Norway; Norwegian Competence Center for Sleep Disorders, Haukeland University Hospital, Bergen, Norway; Norwegian Competence Center for Sleep Disorders, Haukeland University Hospital, Bergen, Norway; Department of Psychosocial Science, University of Bergen, Bergen, Norway; Norwegian Competence Center for Sleep Disorders, Haukeland University Hospital, Bergen, Norway; Department of Psychosocial Science, University of Bergen, Bergen, Norway; Department of Psychiatry, University of Pittsburgh School of Medicine, Pittsburgh, USA; Norwegian Competence Center for Sleep Disorders, Haukeland University Hospital, Bergen, Norway

**Keywords:** RU_SATED, multidimensional sleep health, insomnia, evening type, morning type

## Abstract

**Objectives:**

The aims were to explore multidimensional sleep health and the different dimensions of sleep health in the adult Norwegian population in relation to sex, age, education, circadian preference, and chronic insomnia.

**Methods:**

A representative sample of 1028 Norwegians, aged 18 + years completed a cross-sectional web-based survey. Sleep health was measured with the multidimensional RU_SATED scale, which assesses the dimensions of regularity, satisfaction, alertness, timing, efficiency, and duration. Insomnia was assessed with the Bergen Insomnia Scale. Data were analyzed with chi-square tests, *t*-tests, one-way ANOVAs, and regression analyses, as appropriate. Response rate was 33.5%.

**Results:**

Sleep health was better in males, with increasing age, and with higher educational level, and was poorer in participants with evening preference and chronic insomnia, compared to their respective counterparts. When investigating the different sleep health dimensions, males scored better than females on satisfaction (adjusted odds ratio [aOR] = 0.69, 95% CI = 0.51 to 0.93), timing (aOR = 0.66, 95% CI = 0.49 to 0.88), and efficiency (aOR = 0.68, 95% CI = 0.52 to 0.89). Older age was associated with better scores on regularity and satisfaction, whereas young age was associated with better scores on alertness and duration. High educational level was associated with better scores on alertness, timing, and duration. Evening types scored worse than morning types on regularity (aOR = 0.27, 95% CI = 0.18 to 0.41), satisfaction (aOR = 0.37, 95% CI = 0.26 to 0.53), and timing (aOR = 0.36, 95% CI = 0.26 to 0.51). Participants with chronic insomnia scored worse than participants without insomnia on all six sleep health dimensions.

**Conclusions:**

Sleep health differed significantly in relation to sex, age, education, circadian preference, and chronic insomnia. However, specific group differences were not equally evident in all sleep health dimensions.

**State of Significance**

Sleep health may be conceptualized as a multidimensional pattern of sleep–wakefulness that promotes physical and mental well-being. The construct is different from absence–presence of a sleep disorder, and can be defined in terms of regularity, subjective satisfaction, alertness, timing, efficiency, and sleep duration. Few studies have assessed how multidimensional sleep health and its different dimensions are associated with sex, age, educational level, and circadian preference. Although people with sleep disorders such as insomnia would be expected to have poor sleep health, it is unclear which specific dimensions are affected. This study assessed sleep health and its multiple dimensions in a representative sample of Norwegian adults. The findings add important information and nuance to factors that influence multidimensional sleep health.

## Introduction

Sleep health has been conceptualized in a multitude of ways but typically with the inclusion of sleep problems and sleep disorders [1–4]. A decade ago, Buysse et al. provided a new framework for understanding sleep health, defining it as “a multidimensional pattern of sleep-wakefulness, adapted to individual, social, and environmental demands, that promotes physical and mental well-being” [5]. This conceptualization regards sleep health to be something beyond the absence of a disorder, thus differing from previous perspectives which consider sleep health in terms of illness or lack of illness [5, 6]. Rather, sleep health can be defined and characterized by multiple features such as high regularity, subjective satisfaction, sustained alertness, appropriate timing, high efficiency, and adequate duration of sleep. This definition of sleep health renders it independent of any specific sleep problem, and it can be measured and quantified in individuals both with or without sleep disorders [5].

The scale RU_SATED v2.0 was developed to enable assessment of the various dimensions of sleep health that have been shown to be associated with a range of health outcomes, including physical functioning, metabolic disorders, cardiovascular diseases, and mortality [5, 7, 8]. The six sleep health dimensions assessed by this scale include regularity, satisfaction, alertness, timing, efficiency, and duration. So far only a few studies have used this scale to measure sleep health in the general adult population. One study, conducted during the coronavirus disease-2019 (COVID-19) pandemic, showed that older age, being partnered, and living in a higher-income country were associated with better sleep health, whereas pandemic-related factors such as being laid off from work, financial strain, and strict level of quarantine were associated with poorer sleep health [9]. Poor sleep health was also associated with anxiety and depression. Another study using RU_SATED also showed that older age was associated with better sleep health, and in addition, this study showed that daily TV, social media, or internet use were associated with poorer sleep health [6]. The scale has also been translated and validated in French [10] and in Spanish [11]. In the French study, post hoc analyses showed that even among individuals without sleep and sleepiness problems, the score on the RU_SATED had strong association with health outcomes. This suggests that sleep health remains an important health determinant, also in individuals without sleep disorders [10]. In an actigraphy factor analysis study, similar sleep health domains as those included in RU_SATED were identified, supporting the usefulness of this self-report scale [12].

Considering that many sleep disorders tend to increase with increasing age, it is somewhat surprising that these two earlier studies [6, 9] showed better sleep health in older versus younger individuals. We believe this underscores the important distinction between sleep health and sleep problems. One may have severe sleep problems, but still follow sleep hygiene advice with high regularity and timing of the sleep period, and both are factors that will increase the sleep health score. Of note, the two studies which showed better sleep health in older individuals, investigated the total sleep health (RU_SATED) score. However, since sleep health is measured by several dimensions, age may be associated with the dimensions in a differential manner. One study showed that self-reported sleep disturbance and tiredness declined across the lifespan [13]. On the other hand, older age is associated with reduced amount of slow-wave sleep [14], and the dimension of sleep satisfaction may therefore be rated lower by increasing age. With regards to sex, females report more insomnia symptoms than males [15, 16], possibly suggesting that sleep health may be poorer in females. However, other sleep disorders, such as obstructive sleep apnea, are more common in males [17]. Whether sleep health as measured by regularity, satisfaction, alertness, timing, efficiency, and duration differs between the sexes, is more uncertain. A recent study reported no sex differences in the total sleep health score [9], but more studies are warranted. Furthermore, no studies have so far examined how sleep health is associated with educational level. Insomnia symptoms are more common among individuals with lower compared to higher educational levels [15], but whether sleep health also differs is not known.

Many studies show that evening types suffer from more sleep problems and circadian disturbance than morning types [18]. Thus, evening types may have overall poorer sleep health than morning types, but how the different sleep health dimensions are associated with circadian preference is unclear. To the best of our knowledge, no studies have examined how multidimensional sleep health is associated with circadian preference.

Chronic insomnia is common in the general adult population [16, 19]. It is expected that individuals with insomnia report poorer sleep health than individuals without insomnia, since poor satisfaction with sleep and low efficiency are typical symptoms of insomnia. Furthermore, the timing dimension in RU_SATED is likely related to insomnia, as evening types report more insomnia symptoms than morning types [18]. Still, not all dimensions of sleep health may be associated with insomnia. Individuals with insomnia may for instance try to reduce their complaints by being more regular in their sleep patterns, compared to individuals not complaining of insomnia, who may be able to sleep well without following specific sleep hygiene advice [20]. However, intra-individual variability in sleep patterns has been found to be positively associated with insomnia symptoms [21, 22], which makes it conceivable that scores also on the sleep health dimension regularity may be worse among participants with insomnia.

Against this background, the aims of the present study were to examine both multidimensional sleep health and the different dimensions of sleep health in the general adult population, and study possible associations with sex, age, educational level, circadian preference, and chronic insomnia. In line with earlier research, we hypothesized better total sleep health in old versus young age and in high versus low educational level, and poorer sleep health in individuals with an evening versus morning circadian preference, and in individuals with versus without chronic insomnia. With regards to sex, one previous study showed no differences, letting us to expect similar sleep health in males and females. In terms of the six different dimensions of sleep health, no specific hypotheses were made, thus we aimed to explore their relationships with sex, age, educational level, circadian preference, and chronic insomnia, respectively.

## Participants and Methods

### Design and study population

A representative population-based sample of Norwegian adults aged 18 years or older were invited to participate in a cross-sectional web-based survey on sleep and health. The survey was technically administered by the opinion research institute Kantar TNS and the sample was selected from Kantar’s web panel (see ethics section below). This panel is designed to reflect the demography of the general Norwegian population. Data were collected during April and May 2022. The goal was to include at least 1000 participants. Out of 3071 participants randomly selected by sex, age, and geography, a total of 1028 responded to the study, yielding a response rate of 33.5%.

### Sociodemographic variables and circadian preference

We collected data on sex (male, female), age (in this study grouped into the following categories: 18–29 years, 30–44 years, 45–59 years, 60 years and older), educational level (grouped into primary school, secondary school, and college/university), and circadian preference. Circadian preference was assessed by a single question: “Are you a morning- or an evening person?,” with the following response options: “I am very alert and active in the morning, and sleepy early in the evening (definitely a morning person)”; “I am rather alert in the morning and rather sleepy in the evening (more morning- than evening person)”; “Neither morning- nor evening person”; “I am rather alert in the evening and rather sleepy in the morning (more evening- than morning person)”; “I am very alert and active in the evening and sleepy in the morning (definitely an evening person).” Circadian preference was grouped into three categories: morning type (first two response options), intermediate type, and evening type (last two response options). This item was adapted from the Horne-Ösberg Morningness–Eveningness Questionnaire [23].

### Insomnia

Insomnia symptoms were assessed with the Bergen Insomnia Scale (BIS) [24]. BIS consists of six items, and was developed based on the diagnostic criteria for insomnia according to the Diagnostic and Statistical Manual for Mental Disorders version 4 (DSM-IV-TR) [25]. The items are scored along an eight-point scale indicating the number of days per week for which a specific insomnia symptom is experienced (0–7 days). The items refer to sleep onset (sleep latency exceeding 30 minutes), wake after sleep onset (more than 30 minutes), early morning awakening (more than 30 minutes), non-restorative sleep, daytime impairment, and dissatisfaction with sleep. The BIS originally specified insomnia symptoms experienced during the past month, but this was modified in the present study to a time frame of last 3 months in accordance with the updated DSM-5/International Classification of Sleep Disorders-3 (ICSD-3) diagnostic criteria [26, 27]. According to the DSM-5/ICSD-3 criteria, chronic insomnia, based on the modified BIS, was defined as scoring 3 days per week or more on at least one of the first three items, as well as 3 days per week or more on at least one of the latter two items. The scale has acceptable test–retest reliability, and good convergent and discriminative validity in relation to other self-report measures as well as to polysomnographic data [24]. Cronbach’s alpha for the BIS was 0.81 in the present sample.

### Sleep health

Sleep health was assessed with the validated multidimensional RU_SATED v2.0 scale [5]. For the purpose of the present study, RU_SATED v2.0 was translated from English to Norwegian, using combined translational methods (independent translation by three translators followed by back-translation). The RU_SATED v2.0 scale assesses six dimensions of sleep during the past 1 month. Regularity: “Do you go to bed and get out of bed at about the same time (within one hour) every day?”; satisfaction: “Are you satisfied with your sleep?”; alertness: “Do you stay awake all day without dozing?”; timing: “Is the middle of your sleep between 2:00 a.m. and 4:00 a.m.?”; efficiency: “Do you spend less than 30 minutes awake at night? This includes the time it takes to fall asleep plus awakenings during sleep.”; duration: “Do you sleep between 6 and 8 hours per day?.” The participants were asked to respond to these six dimensions on a three-point Likert scale with the following response alternatives: 0 = rarely/never; 1 = sometimes; 2 = usually/always. A total score is calculated by adding the score of all dimensions, where higher scores indicate better sleep health [5, 28]. The scale has been reported to have good psychometric properties [28]. As the RU_SATED has some properties in line with a formative scale [29, 30], calculation of internal consistency was not conducted.

### Ethics

Kantar’s web panel is a consent-based database with 40 000 participants recruited to reflect the demography of the Norwegian population. Kantar TNS is data controller for all personal data in the web panel, and their procedures for data collection are in accordance with GDPR (General Data Protection Regulation) within the EU. According to Kantar’s procedures, no personal data are disclosed. Before responding to this specific questionnaire, participants received general information about the purpose of the study, and that anonymous data would be provided to the research team. The potential participants accepted or rejected participation before responding to any specific question. Since only anonymous data were provided to the research team, the survey was exempted from review by the Regional Committee for Medical and Health Related Research Ethics (REK Nord, application number 458482).

### Statistics

Data analyses were conducted with SPSS, version 28 (IBM SPSS Statistics). The data were weighted according to the population distribution of age, sex, and county of living, to correct for potential divergence between the sample and the distribution in the general population. The associations between the total RU_SATED score and sex, age group, educational level, circadian preference, and chronic insomnia were explored using *t*-test for independent samples or one-way ANOVA, as appropriate. Furthermore, a linear regression analysis was conducted with the total RU_SATED score as the dependent variable and sex, age group, educational level, circadian preference, and chronic insomnia as independent variables in a fully adjusted model (in which age group, educational level, and circadian preference were treated as continuous variables). The associations between each of the six individual dimensions of RU_SATED and sex, age group, educational level, circadian preference, and chronic insomnia were explored using Pearson chi-square statistics. Furthermore, crude logistic regression analyses were conducted with each dimension on RU_SATED as dependent variable (never/rarely/sometimes = 0; usually/always = 1) and sex, age group, educational level, circadian preference, and chronic insomnia as categorical covariates. In the adjusted logistic regression analyses, all covariates were entered together in the analysis. Significance level was set to 0.05.

## Results

The characteristics of the study sample are presented in Table 1. In total, 49.6% were females, the different age groups were about equally represented, and a majority reported having a college/university education. In terms of circadian preference, 37.1% of the participants reported being morning types and 35.0% evening types. The prevalence of chronic insomnia was 24.9%.

**Table 1.** Characteristics of the Norwegian Sample (*n* = 1028) and Sleep Health as Measured by the Total Score on the RU_SATED Scale in Relation to Sex, Age Group, Educational Level, Circadian Preference, and Chronic Insomnia

	% (*n*)	Total RU_SATED score Mean (SD)	*P*-value
All participants	100 (1028)	8.96 (2.10)	
Sex			
Male	50.4 (518)	9.13 (2.11)	*.008*
Female	49.6 (510)	8.78 (2.08)	
Age			
18–29 years	19.6 (201)	8.49 (2.31)	**<** *.001*
30–44 years	25.7 (264)	8.78 (1.94)	
45–59 years	25.7 (264)	9.12 (2.15)	
≥60 years	29.0 (298)	9.30 (2.10)	
Education			
Primary school	4.5 (46)	8.07 (2.67)	**<** *.001*
Secondary school	37.1 (381)	8.67 (2.20)	
College/university	58.4 (600)	9.21 (1.94)	
Circadian preference			
Morning type	37.1 (378)	9.35 (1.91)	**<** *.001*
Intermediate	27.9 (284)	8.98 (2.28)	
Evening type	35.0 (357)	8.55 (2.05)	
Chronic insomnia			
No	75.1 (769)	9.48 (1.89)	**<** *.001*
Yes	24.9 (255)	7.35 (1.92)	

Total score is the sum of all sleep health dimensions (range 0–12). Significant results are indicated in italics. Data are weighted for sex, age, and county of living.

Average total sleep health score for the whole sample was 8.96 (SD 2.10) (Table 1). Sleep health was significantly better in males and with increasing age as compared to females and younger age, respectively. Furthermore, higher educational level was associated with better sleep health. For circadian preference, morning types reported best sleep health whereas evening types reported the poorest. Participants with chronic insomnia reported poorer sleep health as compared to participants without insomnia (Table 1).

Linear regressions with the total sleep health score as dependent variable and with all predictors included in the same analysis, showed that age group (*B* = 0.15, std.error = 0.05, *β* = 0.08, *t* = 2.72, *p* = .007) and educational level (*B* = 0.61, std.error = 0.10, *β* = 0.17, *t* = 6.09, *p* < .001) were positively associated with sleep health. On the other hand, evening circadian preference (*B* = −0.36, std.error = 0.07, *β* = −0.14, *t* = −5.13, *p* < .001) and chronic insomnia (*B* = −2.00, std.error = 0.14, *β* = −0.14, *t* = −5.13, *p* < .001) were negatively associated with sleep health. Sex (*B* = −0.22, std.error = 0.12, *β* = −0.05, *t* = −1.87, *p* = .061) was not significantly associated with total sleep health score in the fully adjusted model.

Table 2 shows the responses to the different sleep health dimensions in the whole sample and in relation to sex and age group. For the whole sample, the percentage reporting the highest score (responding usually/always) on the different sleep health dimensions ranged from 76.1% on the regularity to 35.1% on the efficiency dimension (Table 2).

**Table 2.** Data From the Different Sleep Health Dimensions Among the Whole Sample, and in Relation to Sex and Age (*n* = 1028)

	All, % (*n*)	Sex, % (*n*)			Age, % (*n*)				
		Male	Female	*P*-value	18–29 years	30–44 years	45–59 years	60 + years	*P*-value
Regularity									
Rarely/never	5.4 (56)	6.0 (31)	4.7 (24)	.241	14.9 (30)	3.8 (10)	3.8 (10)	1.7 (5)	**<** *.001*
Sometimes	18.5 (190)	20.1 (104)	16.9 (86)		26.4 (53)	26.4 (70)	13.3 (35)	11.1 (33)	
Usually/always	76.1 (782)	73.9 (383)	78.4 (399)		58.7 (118)	69.8 (185)	83.0 (219)	87.2 (260)	
Satisfaction									
Rarely/never	12.6 (129)	10.8 (56)	14.4 (73)	*.001*	16.3 (33)	13.7 (36)	14.4 (38)	7.4 (22)	**<** *.001*
Sometimes	37.7 (386)	33.8 (175)	41.5 (211)		42.6 (86)	46.4 (122)	37.3 (98)	26.9 (80)	
Usually/always	49.8 (510)	55.3 (286)	44.1 (224)		41.1 (83)	39.9 (105)	48.3 (127)	65.7 (195)	
Alertness									
Rarely/never	9.0 (92)	10.1 (52)	7.9 (40)	.329	6.5 (13)	7.9 (21)	9.1 (24)	11.5 (34)	*.011*
Sometimes	22.9 (235)	23.8 (123)	22.1 (112)		21.9 (44)	18.5 (49)	21.3 (56)	29.2 (86)	
Usually/always	68.1 (697)	66.2 (342)	70.0 (355)		71.6 (144)	73.6 (195)	69.6 (183)	59.3 (175)	
Timing									
Rarely/never	9.7 (99)	8.7 (45)	10.6 (54)	*.006*	16.0 (32)	8.7 (23)	8.0 (21)	8.1 (24)	*.003*
Sometimes	21.5 (220)	18.0 (93)	25.2 (128)		23.5 (47)	26.4 (70)	18.9 (50)	18.2 (54)	
Usually/always	68.8 (705)	73.3 (378)	64.2 (326)		60.5 (121)	64.9 (172)	73.1 (193)	73.6 (218)	
Efficiency									
Rarely/never	27.3 (279)	25.1 (130)	29.4 (149)	*.004*	32.0 (64)	27.7 (73)	23.3 (61)	27.3 (81)	*.019*
Sometimes	37.6 (385)	34.8 (180)	40.5 (205)		39.5 (79)	39.0 (103)	32.4 (85)	39.7 (118)	
Usually/always	35.1 (359)	40.0 (207)	30.0 (152)		28.5 (57)	33.3 (88)	44.3 (116)	33.0 (98)	
Duration									
Rarely/never	7.4 (76)	6.6 (34)	8.3 (42)	.186	4.5 (9)	6.5 (17)	10.2 (27)	7.8 (23)	*.003*
Sometimes	23.4 (239)	21.7 (112)	25.2 (128)		19.8 (40)	30.0 (79)	25.4 (67)	18.0 (53)	
Usually/always	69.2 (708)	71.8 (371)	66.5 (338)		75.7 (153)	63.5 (167)	64.4 (170)	74.1 (218)	

Regularity: Do you go to bed and get out of bed at about the same time (within one hour) every day? Satisfaction: Are you satisfied with your sleep? Alertness: Do you stay awake all day without dozing? Timing Is the middle of your sleep between 2:00 a.m. and 4:00 a.m.? Efficiency: Do you spend less than 30 minutes awake at night? This includes the time it takes to fall asleep plus awakenings during sleep. Duration: Do you sleep between 6 and 8 hours per day? Significant results from the chi-square tests are indicated in italics. Data are weighted for sex, age, and county of living.

When examining the individual sleep health dimensions, males had better scores than females on satisfaction, timing, and efficiency, whereas no sex differences were detected for regularity, alertness, or duration (Table 2). With regards to age group, higher age was associated with better scores on regularity, satisfaction, and timing, whereas higher age was associated with worse scores on alertness. For efficiency, the 45–59-year-old group scored better than the other age groups. For duration, both the youngest (18–29 years) and oldest (≥60 years) age groups had better scores than the other age groups (Table 2).

Table 3 depicts the responses to the different sleep health dimensions in relation to educational level, circadian preference, and chronic insomnia. Participants with college/university education had significantly better scores on regularity, timing, and duration, whereas no differences in relation to educational level were found for the other sleep health dimensions. For circadian preference, morning types had better scores on regularity, satisfaction, and timing. No significant differences in relation to circadian preference were found on alertness, efficiency, or duration (Table 3). Participants with chronic insomnia had worse scores than participants without insomnia on all the individual sleep health dimensions. The difference between participants with versus without chronic insomnia was largest for the dimension Satisfaction (usually/always: 7.9% vs. 63.9%).

**Table 3.** Data From the Different Sleep Health Dimensions in Relation to Education, Circadian Preference, and Chronic Insomnia Disorder (*n* = 1028)

	Education, % (*n*)				Circadian preference, % (*n*)				Chronic insomnia, % (*n*)		
	Primary school	Secondary school	College/ university	*P*-value	Morning type	Intermediate	Evening type	*P*-value	No	Yes	*P*-value
Regularity											
Rarely/never	10.6 (5)	7.3 (28)	3.8 (23)	*.009*	1.6 (6)	6.4 (18)	8.4 (30)	**<** *.001*	4.0 (31)	9.8 (25)	*.001*
Sometimes	17.0 (8)	21.8 (83)	16.6 (100)		10.1 (38)	19.1 (54)	27.5 (98)		17.9 (138)	19.6 (50)	
Usually/always	72.3 (34)	70.9 (270)	79.5 (478)		88.4 (334)	74.6 (211)	64.1 (229)		78.0 (600)	70.6 (180)	
Satisfaction											
Rarely/never	19.6 (9)	14.8 (56)	10.5 (63)	.137	9.3 (35)	13.4 (38)	15.2 (54)	**<** *.001*	4.2 (32)	37.7 (95)	**<** *.001*
Sometimes	28.3 (13)	37.5 (142)	38.5 (230)		31.1 (117)	35.9 (102)	46.6 (166)		31.9 (245)	54.4 (137)	
Usually/always	52.2 (24)	47.8 (181)	51.0 (305)		59.6 (224)	50.7 (144)	38.2 (136)		63.9 (490)	7.9 (20)	
Alertness											
Rarely/never	13.0 (6)	8.7 (33)	8.9 (92)	.093	9.6 (36)	10.6 (30)	7.5 (27)	.365	8.2 (63)	11.4 (29)	*.007*
Sometimes	34.8 (16)	25.0 (95)	20.8 (124)		23.7 (89)	24.1 (68)	20.4 (73)		20.9 (160)	28.3 (72)	
Usually/always	52.2 (24)	66.3 (252)	70.4 (420)		66.7 (250)	65.2 (184)	72.1 (258)		70.8 (542)	60.2 (153)	
Timing											
Rarely/never	15.2 (7)	11.8 (45)	8.0 (48)	*.007*	8.0 (30)	6.4 (18)	13.8 (49)	**<** *.001*	8.8 (68)	12.3 (31)	**<** *.001*
Sometimes	34.8 (16)	22.9 (87)	19.7 (118)		14.1 (53)	20.1 (57)	30.1 (107)		17.4 (134)	33.7 (85)	
Usually/always	50.0 (23)	65.3 (248)	72.3 (433)		77.9 (293)	73.5 (208)	56.1 (199)		73.7 (567)	54.0 (136)	
Efficiency											
Rarely/never	39.1 (18)	27.9 (105)	25.8 (155)	.100	29.7 (112)	24.9 (70)	26.8 (95)	.609	25.1 (193)	33.6 (85)	**<** *.001*
Sometimes	28.3 (13)	40.7 (153)	36.5 (219)		36.1 (136)	40.2 (113)	36.3 (129)		36.1 (277)	42.3 (107)	
Usually/always	32.6 (15)	31.4 (118)	37.7 (226)		34.2 (129)	34.9 (98)	36.9 (131)		38.8 (298)	24.1 (61)	
Duration											
Rarely/never	17.0 (8)	9.8 (37)	5.0 (30)	*.001*	6.4 (24)	7.8 (22)	8.7 (31)	.259	4.7 (36)	15.9 (40)	**<** *.001*
Sometimes	21.3 (10)	25.9 (98)	22.1 (132)		20.5 (77)	23.0 (65)	26.1 (93)		16.6 (128)	44.0 (111)	
Usually/always	61.7 (29)	64.4 (244)	72.9 (435)		73.1 (274)	69.3 (196)	65.3 (233)		78.7 (605)	40.1 (101)	

Regularity: Do you go to bed and get out of bed at about the same time (within one hour) every day? Satisfaction: Are you satisfied with your sleep? Alertness: Do you stay awake all day without dozing? Timing: Is the middle of your sleep between 2:00 a.m. and 4:00 a.m.? Efficiency: Do you spend less than 30 minutes awake at night? This includes the time it takes to fall asleep plus awakenings during sleep. Duration: Do you sleep between 6 and 8 hours per day? Significant results from the chi-square tests are indicated in italics. Data are weighted for sex, age, and county of living.

Table 4 shows crude and adjusted logistic regression analyses with the different sleep health dimensions as dependent variables. In line with the results from the chi-square tests, males had significantly better scores on satisfaction, timing, and efficiency in the adjusted analyses. Higher age was associated in linear fashion with better scores on regularity, with an adjusted odds ratio of 4.39 (95% CI = 2.76 to 6.99) for participants ≥60 years (with 18–29 years as reference). The oldest participants (≥60 years) also had significantly better scores on satisfaction, but worse scores on alertness (Table 4). For timing and efficiency, only participants aged 45–59 years had significantly better scores in the adjusted analyses. For duration, all age groups had worse scores than the reference group aged 18–29 years (Table 4). For educational level, the adjusted analyses showed that the college/university group had better scores on alertness, timing, and duration, whereas the secondary school group only had better scores than the primary school group on timing. Adjusted analyses showed circadian preference associations with regularity, satisfaction, and timing, with best scores among morning types (Table 4). For chronic insomnia, the analyses showed worse scores on all the different sleep health dimensions, but the score on regularity was not significant in the adjusted analyses.

**Table 4.** Logistic Regression Analyses With the Different Dimensions of Sleep Health (Never/Rarely/Sometimes = 0; Usually/Always = 1) as the Dependent Variable and Sociodemographic Variables, Circadian Preference, and Chronic Insomnia as Predictors Among a Representative Sample of Norwegian Adults (*n* = 1028). In the Adjusted Model, all Predictors Were Entered in the Same Analysis

	Crude	Adjusted	Crude	Adjusted	Crude	Adjusted
	Regularity, OR (95% CI)		Satisfaction, OR (95% CI)		Alertness, OR (95% CI)	
Sex						
Male	1.00 (ref)	1.00 (ref)	1.00 (ref)	1.00 (ref)	1.00 (ref)	1.00 (ref)
Female	1.28 (0.96 to 1.71)	1.35 (0.99 to 1.86)	*0.64 (0.50 to 0.81)*	*0.69 (0.51 to 0.93)*	1.19 (0.92 to 1.55)	1.22 (0.93 to 1.61)
Age group						
18–29 years	1.00 (ref)	1.00 (ref)	1.00 (ref)	1.00 (ref)	1.00 (ref)	1.00 (ref)
30–44 years	*1.64 (1.12 to 2.41)*	*1.81 (1.20 to 2.73)*	0.96 (0.66 to 1.39)	0.92 (0.60 to 1.42)	1.11 (0.73 to 1.67)	0.99 (0.64 to 1.52)
45–59 years	*3.43 (2.24 to 5.25)*	*3.53 (2.25 to 5.54)*	1.33 (0.92 to 1.92)	1.33 (0.86 to 2.07)	0.90 (0.60 to 1.35)	0.86 (0.56 to 1.30)
≥60 years	*4.80 (3.09 to 7.46)*	*4.39 (2.76 to 6.99)*	*2.75 (1.90 to 3.98)*	*2.29 (1.48 to 3.52)*	*0.58 (0.39 to 0.85)*	*0.54 (0.36 to 0.81)*
Education						
Primary school	1.00 (ref)	1.00 (ref)	1.00 (ref)	1.00 (ref)	1.00 (ref)	1.00 (ref)
Secondary school	0.89 (0.45 to 1.77)	0.99 (0.47 to 2.08)	0.85 (0.46 to 1.56)	0.91 (0.44 to 1.89)	1.81 (0.98 to 3.34)	1.67 (0.88 to 3.17)
College/university	1.42 (0.72 to 2.81)	1.67 (0.79 to 3.50)	0.97 (0.53 to 1.76)	1.16 (0.57 to 2.38)	*2.18 (1.19 to 3.98)*	*1.99 (1.06 to 3.74)*
Circadian preference						
Morning type	1.00 (ref)	1.00 (ref)	1.00 (ref)	1.00 (ref)	1.00 (ref)	1.00 (ref)
Intermediate type	*0.38 (0.25 to 0.58)*	*0.40 (0.26 to 0.61)*	*0.70 (0.51 to 0.95)*	*0.60 (0.41 to 0.87)*	0.95 (0.69 to 1.32)	0.95 (0.68 to 1.32)
Evening type	*0.24 (0.16 to 0.34)*	*0.27 (0.18 to 0.41)*	*0.42 (0.31 to 0.56)*	*0.37 (0.26 to 0.53)*	1.31 (0.95 to 1.79)	1.19 (0.86 to 1.65)
Chronic insomnia						
No	1.00 (ref)	1.00 (ref)	1.00 (ref)	1.00 (ref)	1.00 (ref)	1.00 (ref)
Yes	*0.68 (0.49 to 0.93)*	0.74 (0.52 to 1.06)	*0.05 (0.03 to 0.08)*	*0.04 (0.03 to 0.07)*	*0.62 (0.46 to 0.84)*	*0.57 (0.42 to 0.78)*

	Timing, OR (95% CI)		Efficiency, OR (95% CI)		Duration, OR (95% CI)	
Sex						
Male	1.00 (ref)	1.00 (ref)	1.00 (ref)	1.00 (ref)	1.00 (ref)	1.00 (ref)
Female	*0.66 (0.50 to 0.86)*	*0.66 (0.49 to 0.88)*	*0.64 (0.50 to 0.83)*	*0.68 (0.52 to 0.89)*	0.78 (0.60 to 1.02)	0.86 (0.64 to 1.16)
Age group						
18–29 years	1.00 (ref)	1.00 (ref)	1.00 (ref)	1.00 (ref)	1.00 (ref)	1.00 (ref)
30–44 years	1.21 (0.83 to 1.77)	1.15 (0.77 to 1.73)	1.25 (0.84 to 1.87)	1.17 (0.77 to 1.76)	*0.56 (0.37 to 0.84)*	*0.43 (0.27 to 0.68)*
45–59 years	*1.79 (1.21 to 2.66)*	*1.58 (1.04 to 2.41)*	*2.00 (1.35 to 2.96)*	*1.91 (1.28 to 2.87)*	*0.58 (0.38 to 0.87)*	*0.46 (0.29 to 0.72)*
≥60 years	*1.81 (1.23 to 2.65)*	1.33 (0.88 to 2.00)	1.24 (0.84 to 1.83)	1.12 (0.75 to 1.68)	0.91 (0.60 to 1.38)	*0.58 (0.36 to 0.91)*
Education						
Primary school	1.00 (ref)	1.00 (ref)	1.00 (ref)	1.00 (ref)	1.00 (ref)	1.00 (ref)
Secondary school	*1.89 (1.02 to 3.49)*	*2.13 (1.10 to 4.13)*	0.94 (0.49 to 1.81)	0.85 (0.44 to 1.66)	1.10 (0.59 to 2.07)	1.42 (0.71 to 2.83)
College/university	*2.61 (1.43 to 4.77)*	*3.20 (1.67 to 6.15)*	1.24 (0.66 to 2.35)	1.19 (0.62 to 2.28)	1.63 (0.88 to 3.04)	*2.28 (1.15 to 4.53)*
Circadian preference						
Morning type	1.00 (ref)	1.00 (ref)	1.00 (ref)	1.00 (ref)	1.00 (ref)	1.00 (ref)
Intermediate type	0.79 (0.55 to 1.13)	0.80 (0.55 to 1.16)	1.04 (0.75 to 1.43)	1.04 (0.75 to 1.46)	0.82 (0.59 to 1.16)	0.82 (0.57 to 1.19)
Evening type	*0.37 (0.27 to 0.51)*	*0.36 (0.26 to 0.51)*	1.13 (0.83 to 1.52)	1.13 (0.82 to 1.55)	*0.69 (0.50 to 0.95)*	0.71 (0.50 to 1.01)
Chronic insomnia						
No	1.00 (ref)	1.00 (ref)	1.00 (ref)	1.00 (ref)	1.00 (ref)	1.00 (ref)
Yes	*0.42 (0.31 to 0.56)*	*0.45 (0.33 to 0.62)*	*0.50 (0.36 to 0.69)*	*0.52 (0.37 to 0.72)*	*0.18 (0.13 to 0.25)*	*0.19 (0.14 to 0.26)*

CI, confidence interval. Significant results are indicated in italics. Data are weighted for sex, age, and county of living.

## Discussion

Self-reported sleep health was better in males and among individuals with older age and higher education. Conversely, self-reported sleep health was poorer among individuals with evening preference and chronic insomnia, compared with their respective counterparts. These findings were in line with our hypotheses. However, group differences were not present in all the different dimensions of sleep health. These findings add important information and nuance to factors that influence multidimensional sleep health.

The total sleep health score in our sample was 8.96. This was higher than reported in a US study among 3401 participants, in which the mean score was 7.58 [28]. The US study recruited participants via the online platform Amazon’s Mechanical Turk. Whether this may lead to a biased sample is unclear. A score of 8.2 was found in a study among all professionals working at a hospital in France [10]. An online survey with participants from 59 countries across five continents at the beginning of the COVID-19 pandemic reported a mean sleep health score of 8.06 [9], also lower than the mean score of the present study. The multinational survey recruited participants using a snowball sampling method and distributed the survey through social media and professional emailing lists, which most likely renders the sample non-representative of the general population. Still, it is expected that there may be differences in sleep health between countries. Yuksel et al. reported that sleep health was significantly poorer in participants from Latin America and the Caribbean (7.79), compared to Europe and Central Asia (8.32), and North America (8.64) [9]. In 2021, the Commonwealth Fund ranked Norway as having the best health care among 11 high-income countries [31]. However, whether such a ranking of health care among countries also has implications for sleep health is unclear.

In the adjusted analyses, males scored better than females on the sleep health dimensions of satisfaction, timing, and efficiency, but no differences were seen between sexes on regularity, alertness, and duration. As females report more insomnia symptoms than males [15], the findings that males reported to be more satisfied with their sleep (satisfaction) and spend less time awake at night (efficiency) seem reasonable. Among females, only 44.1% and 30.0% reported “usually/always” to be satisfied with sleep and spending less than 30 minutes awake at night, respectively. The corresponding figures for males were 55.3% and 40.0%, respectively, indicating that also many males were not sleeping well. Of note, in the fully adjusted linear regression model with the total sleep health score as dependent variable, a nonsignificant difference (*p* = .061) between the sexes was found, suggesting that any effect of sex would be small in magnitude. Similarly, no association between total sleep health score and sex was present in the multinational study conducted during the beginning of the COVID-19 pandemic [9].

Our findings corroborated previous studies showing that higher age was associated with better total sleep health [6, 9]. This underscores the important distinction between sleep health and sleep problems, as most sleep problems are more common with age. When studying the different sleep health dimensions, a somewhat more complex picture emerged. In the adjusted analyses, the dose-dependent association with age was especially evident for regularity, with older people being more regular in their sleep patterns compared to their younger counterparts. For alertness, an opposite association was found, where the highest age group showed worse scores. This is in line with other studies showing that older people are less alert and more often doze during the day [32–34]. However, studies also show that younger people report to be more sleepy than older people [35–37], in contrast to the present finding. For duration, the youngest age group scored better than the other age groups. This suggests that more participants aged 18–29 years obtained the recommended sleep duration [38]. In his seminal paper, Buysse et al. [5] states that the sleep health dimension satisfaction may correspond with the amount of slow-wave sleep. However, slow-wave sleep is reduced by aging [14, 37], and the finding that older participants scored better than younger participants on this sleep health dimension is not in line with the notion that satisfaction corresponds with slow-wave sleep.

Regarding educational level, higher educational level was associated with better total sleep health. For the different sleep health dimensions, the adjusted analyses showed that college/university education was associated with better scores on alertness, timing, and duration, but not on the other dimensions. Insomnia symptoms are less common in individuals with higher educational levels [15], suggesting that disadvantageous social factors interfere with sleep. However, somewhat surprisingly, the satisfaction dimension was not associated with educational level, as around 50% reported to be “usually/always” satisfied with their sleep, irrespective of education. No other study has investigated sleep health in relation to educational level, but sleep health is not reported to be associated with being employed or not [9].

Circadian preference refers to the preferred timing for sleep and wakefulness, and this preference changes gradually from being an evening type in younger adults towards a morning type in the elderly [39]. Evening circadian preference is associated with short weekday sleep durations (late bedtimes combined with early rise times) and circadian disruption [40]. Studies show that evening types suffer from more sleep problems than morning types [18], and in line with this, we found evening types to score worse than morning types on total sleep health as well as on the dimensions of regularity, satisfaction, and timing. Somewhat surprisingly, alertness and duration were not significantly associated with circadian preference in the adjusted analyses, suggesting that even though evening types reported to be less satisfied with their sleep, this did not seem to affect their ability to stay awake during the daytime or to obtain between 6 to 8 hours of sleep per day.

Participants with chronic insomnia reported poorer sleep health compared with participants without insomnia. Even though this was expected, the definition of sleep health renders it independent of any specific sleep problem, and it can be measured and quantified in individuals both with or without specific sleep disorders [5]. Interestingly, all the different sleep health dimensions, except regularity, were significant in the adjusted analyses. This means that chronic insomnia was associated with less satisfaction with sleep, more problems staying awake during daytime, worse timing of sleep, more time awake at night, more often not obtaining between 6 and 8 hours of sleep per day, but not necessarily irregularity in bed- and rise times. Satisfaction was the dimension showing the largest difference between participants with or without insomnia. These findings corroborate an Australian study showing the sleep health is positively associated with mental well-being [41]. Even though insomnia was clearly associated with poor sleep health, it is important to note the distinction between the two constructs. One may have a sleep problem, but still have e.g. high regularity and appropriate timing of the sleep patterns. This may explain why regularity was not significantly associated with insomnia in our study.

### Strengths and limitations

Our study has several strengths and limitations, as detailed in an earlier paper from the same sample [20]. In brief, one strength was that the participants were randomly drawn from a web panel believed to reflect the general Norwegian population, rendering the sample likely representative and the results generalizable to the adult population in Norway. The response rate was low, but comparable to other similar population-based studies [42]. Another strength was the use of a validated insomnia instrument, the BIS [24]. Other commonly used instruments such as the Pittsburgh Sleep Quality Index and the Insomnia Severity Index could have yielded different results. Moreover, it should be acknowledged that the insomnia diagnosis was based on questionnaire data only, and not a clinical interview. Instruments such as the BIS, and also the Insomnia Severity Index, are not able to exclude the possibility that the insomnia symptoms may be better explained by for instance obstructive sleep apnea or circadian rhythm sleep–wake disorders [43]. Circadian preference was assessed with a single question, and not a valid scale such as the Morningness–Eveningness Questionnaire. Still, this single question is commonly used and expected to accurately categorize individuals into morning and evening types [18]. Unfortunately, data on occupation or shift work were not collected, which is a limitation. Importantly, the cutoffs used in RU_SATED for each sleep health dimension may not apply to all groups across population characteristics, such as age and sex. This needs to be taken into consideration. There were few participants in some of the statistical group analyses, which makes these comparisons problematic to interpret. A major limitation was that all data were based on self-reports and that a cross-sectional design was used, hence factors such as recall bias [44], social desirability bias [45], and the common method bias [46] may have influenced on the findings. Due to the cross-sectional design, no causal interferences can be made in terms of the relationship between the study variables.

In conclusion, sleep health as measured by RU_SATED, was associated with sex, age, educational level, circadian preference, and chronic insomnia. Total sleep health was better in males compared to females, in older compared to younger participants, in participants with higher compared to lower educational levels, in morning compared to evening types, and in participants without chronic insomnia compared to participants with chronic insomnia. However, these group differences were not evident in all the different sleep health dimensions, and for some dimensions, e.g. alertness and age, an inverse association was present.

## Data Availability

The data that support the findings of this study are available from the corresponding author, BB, upon reasonable request.
